# Impact of lateral cortical notching on biomechanical performance in cephalomedullary nailing for unstable pertrochanteric fractures

**DOI:** 10.1007/s00068-024-02596-7

**Published:** 2024-07-17

**Authors:** Sabrina Sandriesser, Niels Ganser, Marianne Hollensteiner, Oliver Trapp, Peter Augat

**Affiliations:** 1 Institute for Biomechanics, BG Unfallklinik Murnau, Prof. Küntscher Str. 8, 82418 Murnau, Germany; 2 Institute for Biomechanics, Paracelsus Medical University, Strubergasse 21, Salzburg, 5020 Austria; 3 Department of Trauma Surgery, BG Unfallklinik Murnau, Prof. Küntscher Str. 8, 82418 Murnau, Germany

**Keywords:** Dynamization, Unstable femur fracture, Cephalomedullary nail, Pertrochanteric fracture, Biomechanics

## Abstract

**Purpose:**

In pertrochanteric femur fractures the risk for fracture healing complications increases with the complexity of the fracture. In addition to dynamization along the lag screw, successful fracture healing may also be facilitated by further dynamization along the shaft axis. The aim of this study was to investigate the mechanical stability of additional axial notch dynamization compared to the standard treatment in an unstable pertrochanteric femur fracture treated with cephalomedullary nailing.

**Methods:**

In 14 human cadaver femora, an unstable pertrochanteric fracture was stabilized with a cephalomedullary nail. Additional axial notch dynamization was enabled in half of the samples and compared against the standard treatment (*n* = 7). Interfragmentary motion, axial construct stiffness and load to failure were investigated in a stepwise increasing cyclic load protocol.

**Results:**

Mean load to failure (1414 ± 234 N vs. 1428 ± 149 N, *p* = 0.89) and mean cycles to failure (197,129 ± 45,087 vs. 191,708 ± 30,490, *p* = 0.81) were equivalent for axial notch dynamization and standard treatment, respectively. Initial construct stiffness was comparable for both groups (axial notch dynamization 684 [593–775] N/mm, standard treatment 618 [497–740] N/mm, *p* = 0.44). In six out of seven specimens the additional axial dynamization facilitated interfragmentary compression, while maintaining its mechanical stability. After initial settling of the constructs, there were no statistically significant differences between the groups for either subsidence or rotation of the femoral head fragment (*p* ≤ 0.30).

**Conclusion:**

Axial notch dynamization provided equivalent mechanical stability compared to standard treatment in an unstable pertrochanteric fracture. Whether the interfragmentary compression generated by axial notch dynamization will promote fracture healing through improved fracture reduction needs to be evaluated clinically.

## Introduction

Trochanteric and subtrochanteric fractures increase with age and osteoporosis, and are therefore most prevalent in elderly women [1, 2]. Subtrochanteric fractures in elderly patients are typically caused by low-energy traumas and most fractures require surgical treatment [3]. Cephalomedullary nails have emerged as the gold standard and the implant of choice for subtrochanteric fractures [3–5]. In more complex fractures involving the lesser trochanter, limited contact area of the fragments typically results in insufficient fracture reduction and lack of interfragmentary compression. In consequence, this leads to healing delays, non-unions or in rare cases even to implant failure [6].

The concept of dynamic hip screws enables dynamization along the femoral neck axis and facilitates improved healing in femoral neck fractures through fracture reduction and interfragmentary compression [7]. As the quality of reduction is one key factor for uneventful fracture healing [8], the concept of dynamization may potentially be beneficial if it would be applicable in more complex trochanteric fractures. Thus, axial dynamization along the shaft axis has been proposed for the treatment of unstable pertrochanteric fractures [6]. This so-called axial notch dynamization is expected to facilitate interfragmentary compression and foster an improved healing process preventing non-unions or implant failures [6, 9, 10]. The superiority of lateral cortical notching in a non-union with fatigue breakage of the cephalomedullary nail was documented in a case report [6]. However, none of these clinical studies evaluated the mechanical performance of additional dynamization along the femoral shaft axis from a biomechanical point of view.

The aim of this study was to investigate the mechanical stability of two different dynamization approaches in an unstable pertrochanteric femoral fracture treated by cephalomedullary nailing. Tapping the lateral cortex below the lag screw offers an innovative option for axial notch dynamization and will be compared against the standard treatment in terms of construct stiffness, interfragmentary motion and load to failure. We hypothesized that axial notch dynamization will provide equivalent or greater maintenance of mechanical stability over time for the fixation of unstable pertrochanteric fractures.

## Materials and methods

Fourteen fresh frozen human femora from female donors older than 60 years were included in this study. Only intact bones without any pathologic deformities or prior implant fixations were accepted. All femora were CT scanned to determine bone mineral density (BMD) by converting the measured Hounsfield units into density (mg/ccm) based on calibration with a phantom (European Forearm Phantom). The center of the femoral head was identified and the slices were aligned perpendicular to the neck axis. Within the cancellous bone structure, two slices in each direction were averaged to determine BMD in the femoral head. The specimens were evenly distributed into the test groups (axial notch dynamization *n* = 7; standard treatment *n* = 7) based on the BMD.

### Specimen preparation

**Fig. 1:**
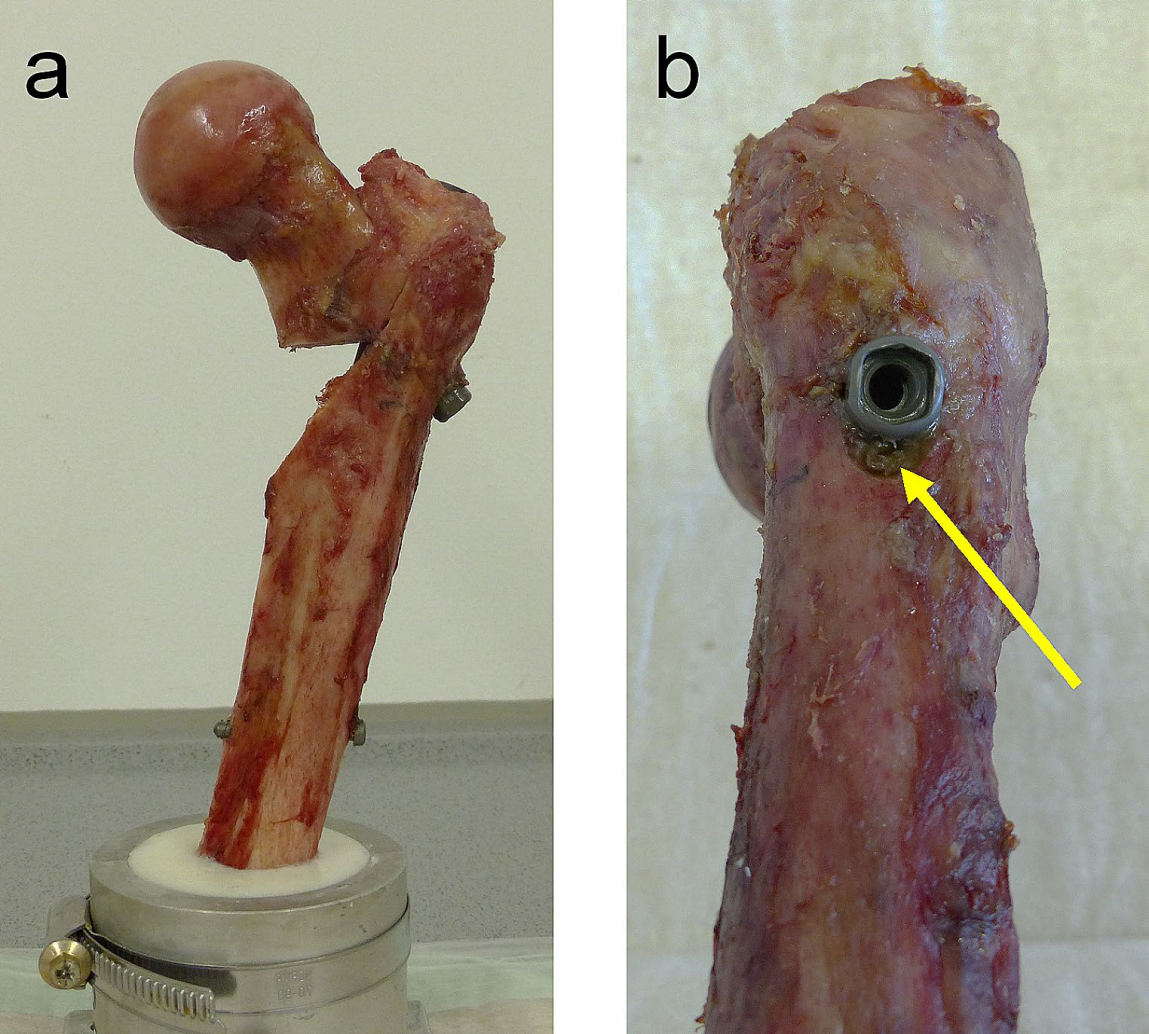
Unstable pertrochanteric fracture (AO/OTA 31A2.2) with a calcar wedge in frontal view **(a)**; axial notch dynamization concept by tapping the lateral cortex indicated by the arrow **(b)**

The specimens were stored at -20° Celsius and thawed overnight before preparation. Soft tissue was dissected and the femora were prepared according to the surgical technique by reaming the medullary canal and by drilling the hole for the lag screw in the intact bone first. Then, using a custom-made sawing template, a series of reproducible proximal femur osteotomies were performed to simulate an unstable pertrochanteric fracture with a lateral wall thickness of the greater trochanter less than 20.5 mm (AO/OTA 31A2.2). The calcar wedge including the lesser trochanter was removed completely (Fig. 1). Additionally, the specimen was cut at 25 cm, measured from the superior aspect of the femoral head.

After osteotomy, implantation of the nail followed (Hansson DCN, 180 mm, 125°, ø 11 mm, Swemac, Sweden). The length of the lag screw (ø 10.75 mm) was measured intraoperatively and lag screw position in the subchondral region was ensured and controlled by radiographs. The set screw was tightened manually according to the manufacturer recommendations and end caps were omitted in this in-vitro study. In the group with axial notch dynamization (*n* = 7), dynamization along the shaft axis was enabled by tapping the lateral cortex below the head of the lag screw according to the manufacturer’s recommendation. Via a guided drill sleeve, the dynamization notch was tapped to the same diameter as the lag screw (Fig. 1b). Distally, the fully threaded locking screw (ø 5 mm) was inserted in the dynamic nail hole and ensured bicortical fixation. After implantation, each bone was aligned at 17° adduction and 11° flexion, to simulate loading at heel strike [11]. Distally the specimens were embedded in an aluminium pot using polyurethane (RenCast FC 53 A/B + filler DT 082, Huntsman; The Woodlands, TX, US).

### Mechanical setup

**Fig. 2:**
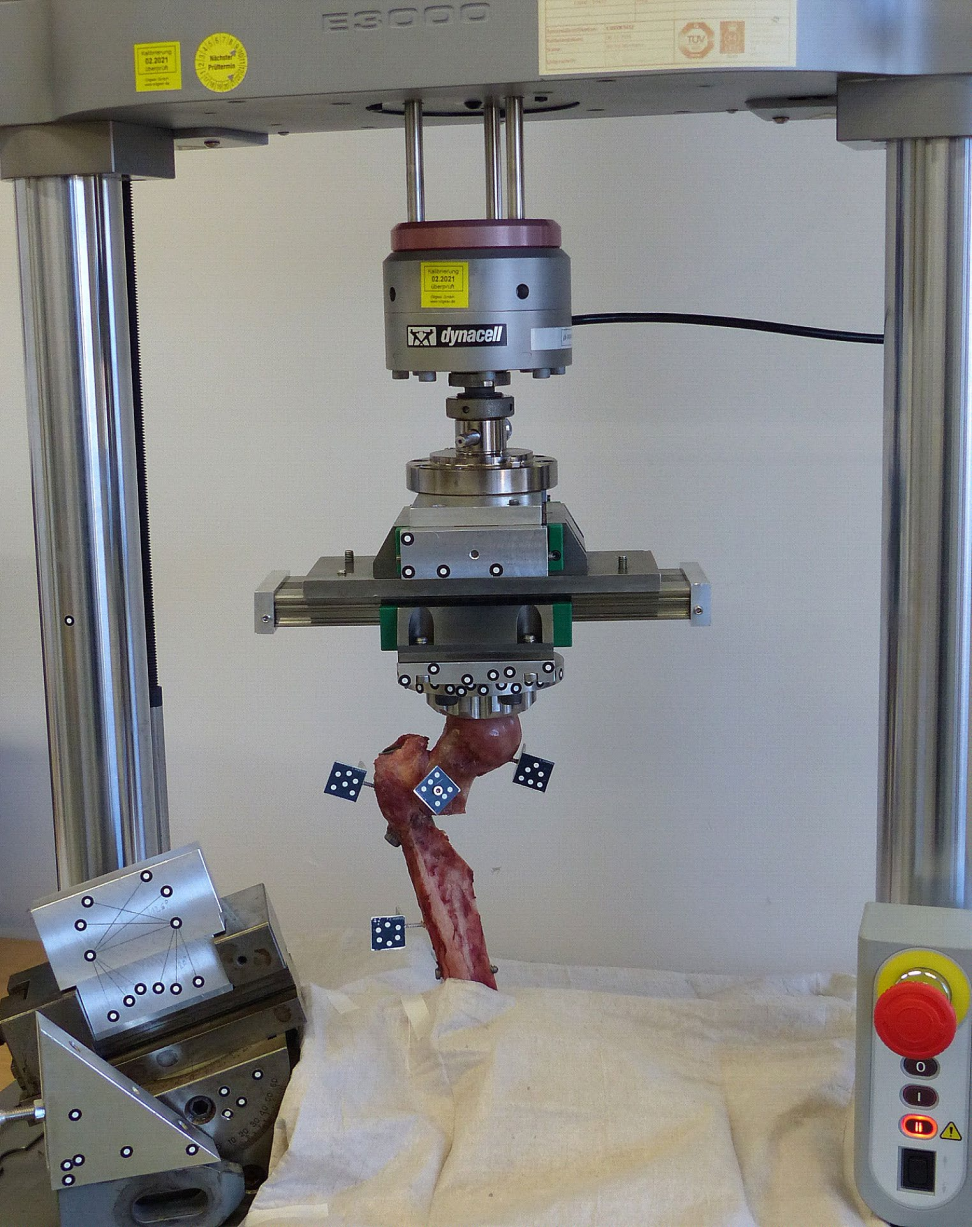
Mechanical loading of the specimen via a conical load applicator attached to a multi-directional bearing plate and the machine actuator. Reference marker clusters defined the alignment of the coordinate system and interfragmentary motion was analyzed based on four marker flags attached to anatomical landmarks

Mechanical tests were conducted on an electrodynamic testing machine (Instron E3000, Instron GmbH, Darmstadt, Germany). Physiological loads were applied on the femoral head via a conical load applicator that was attached to a multidirectional bearing plate and the load cell of the machine actuator (Fig. 2).

Cyclic testing was performed under stepwise increasing axial load until failure. To settle the construct, the specimens were preconditioned for 100 cycles at a sinusoidal load between 50 and 500 N at a frequency of 2 Hz. After that, a quasi-static ramp at a velocity of 0.1 mm/s up to a load of 500 N was conducted to determine initial construct stiffness.

In a next step, cyclic sinusoidal loading at a frequency of 2 Hz started between 50 and 500 N. Every 20,000 cycles, the upper load level increased stepwise by 100 N. At the end of each load level, cyclic loading paused, and the quasi-static stiffness measurement was repeated up to the respective upper load level.

Interfragmentary motion was analyzed with a 3D optical motion tracking system (ARAMIS 5 M, GOM GmbH, Braunschweig, Germany) and pictures were taken at the lower load level of 50 N and the respective maximum load for each load level. To guarantee same marker positions for each specimen, anatomical landmarks were identified. Marker flags were positioned at the femoral head, on both sides along the fracture line and the shaft. Additionally, two reference clusters were installed. The first one represented the coordinate system in space. The second marker cluster defined three coordinate systems and was aligned according to the orientation of the femur (17° adduction / 11° flexion). The first one was in line with the axis of the femoral shaft. The second one was in line with the fracture through the trochanter and the third one was in line with the lag screw. In reference to the respective coordinate system varus collapse, posterior rotation and longitudinal sliding of the head fragment along the fracture line was measured with respect to the rigid greater trochanter fragment.

Cyclic loading was terminated at 20 mm actuator displacement, implying one of the following catastrophic failure modes: varus collapse, excessive subsidence or rotation of the head fragment, bone or implant breakage. Failure load, failure mode, cycles to failure, construct subsidence and head rotations were recorded and analysed.

### Data analysis

Results for construct stiffness and interfragmentary motion were reported as mean values with 95% confidence intervals, and all other results were given as mean ± standard deviation. Subsidence of the head fragment during preconditioning, was measured based on the unloaded states before and after 100 settling cycles. Based on the slope of the linear portion of the load-displacement curve, axial construct stiffness was analysed. After every 20,000 cycles stiffness calculation was repeated. Subsidence of the head fragment along the fracture line, varus collapse and posterior rotation of the head fragment were investigated using the respective coordinate system.

For statistical analysis data were tested for normal distribution using Shapiro Wilk tests (SPSS Statistics, Version 26, IBM, Armonk, NY, USA). BMD and cycles to failure were compared using Mann-Whitney-U tests and further analysed using a Pearson correlation. A Kaplan-Meier survival analysis with log-rank test was applied to evaluate cycles to failure. Axial construct stiffness and interfragmentary motion were compared using unpaired t-tests and analysed at 120,000 load cycles, equivalent to 1100 N, which was the maximum load level of the weakest construct. Level of significance was set to 0.05.

## Results

**Fig. 3:**
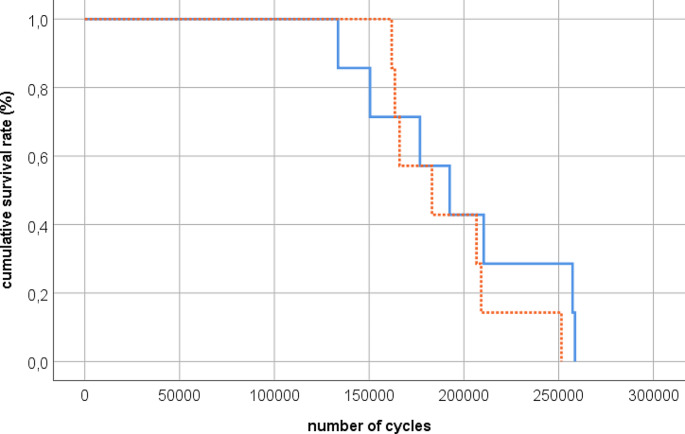
Survival analysis based on cycles to failure for each group (*n* = 7); solid line: axial notch dynamization, dotted line: standard treatment

The specimens were evenly distributed in both study groups based on BMD in the femoral head (axial notch dynamization 187 ± 22 mg/ccm, standard treatment 177 ± 25 mg/ccm, *p* = 0.75). Highest failure load of 1700 N was reached in one specimen in each group, respectively. Mean failure load was comparable for both groups (axial notch dynamization 1414 ± 234 N, standard treatment 1428 ± 149 N, *p* = 0.89). The standard treatment group reached on average 191,708 ± 30,490 cycles to failure, while the axial notch dynamized group reached on average 197,129 ± 45,087 cycles to failure (*p* = 0.81) (Fig. 3). No correlation between BMD and cycles to failure was found (*p* = 0.31).

### Axial construct stiffness

During the initial 100 preconditioning cycles, interfragmentary compression was achieved in six of seven specimens due to additional dynamization along the lateral notch. In comparison to the standard treatment, axial notch dynamization resulted in a significant subsidence of the head fragment along the shaft axis during the preconditioning cycles (3.5 [2.2–4.8] mm vs. 1.2 [0.8–1.7] mm, *p* = 0.001). After this initial settling, both groups showed comparable initial stiffness (axial notch dynamization 684 [593–775] N/mm, standard treatment 618 [497–740] N/mm, *p* = 0.44). Up to 120,000 cycles no significant differences were found between the groups and axial construct stiffness remained on a constant level of approximately 600 N/mm.

### Interfragmentary motion

**Fig. 4:**
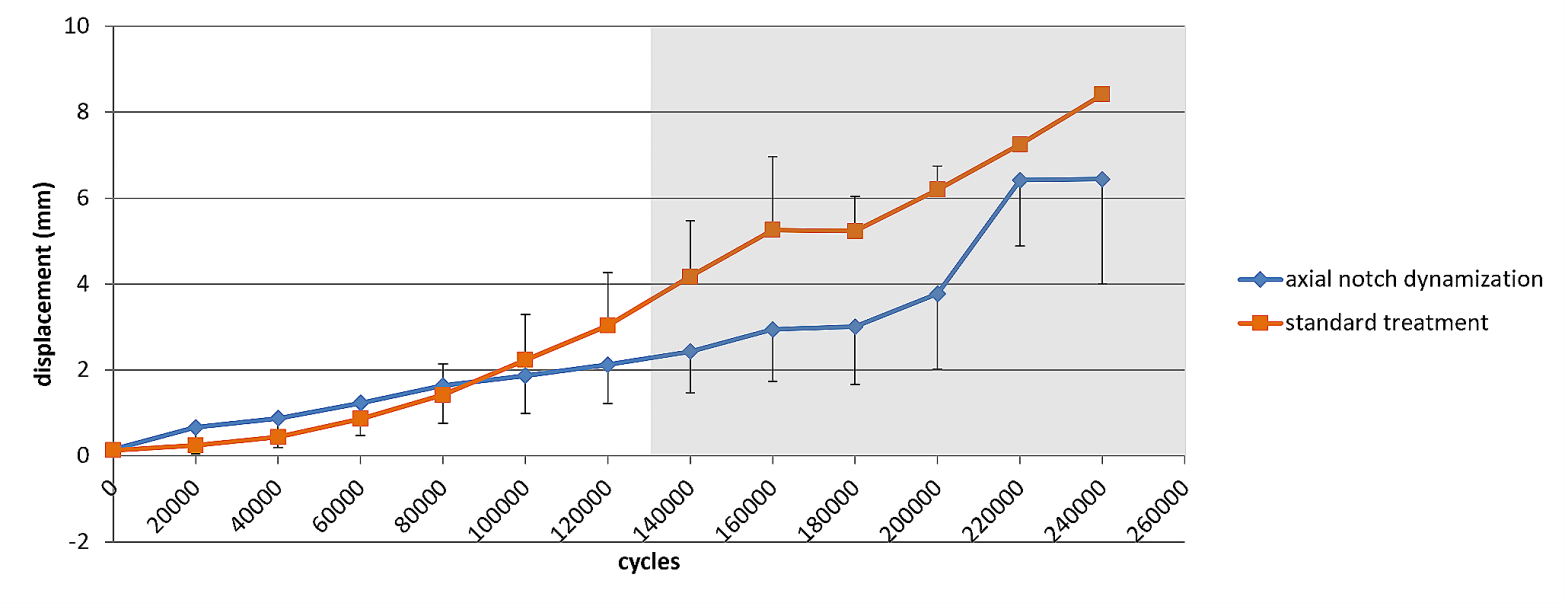
Subsidence of the femoral head fragment along the fracture line (mean with 95% confidence interval). All specimens survived up to 120,000 cycles. For load levels above 1100 N (grey area) only the remaining specimens that survived the respective load level are displayed

Subsidence of the head fragment along the fracture line increased with increasing load levels (Fig. 4). At an axial load of 1100 N (equivalent to 120,000 cycles) the displacement in the standard treatment group was 50% larger compared to the axial notch dynamization group, but this difference was statistically not significant (3.0 [1.8–4.3] mm vs. 2.1 [1.2-3.0] mm, *p* = 0.30).

For varus collapse, both groups showed consistently low values up to a load of 1100 N (axial notch dynamization 2.1° [1.5°-2.8°] vs. standard treatment 1.5° [1.1°-2.0°], *p* = 0.18) and remained at this low level up to 1400 N (equivalent to 180,000 cycles).

**Fig. 5:**
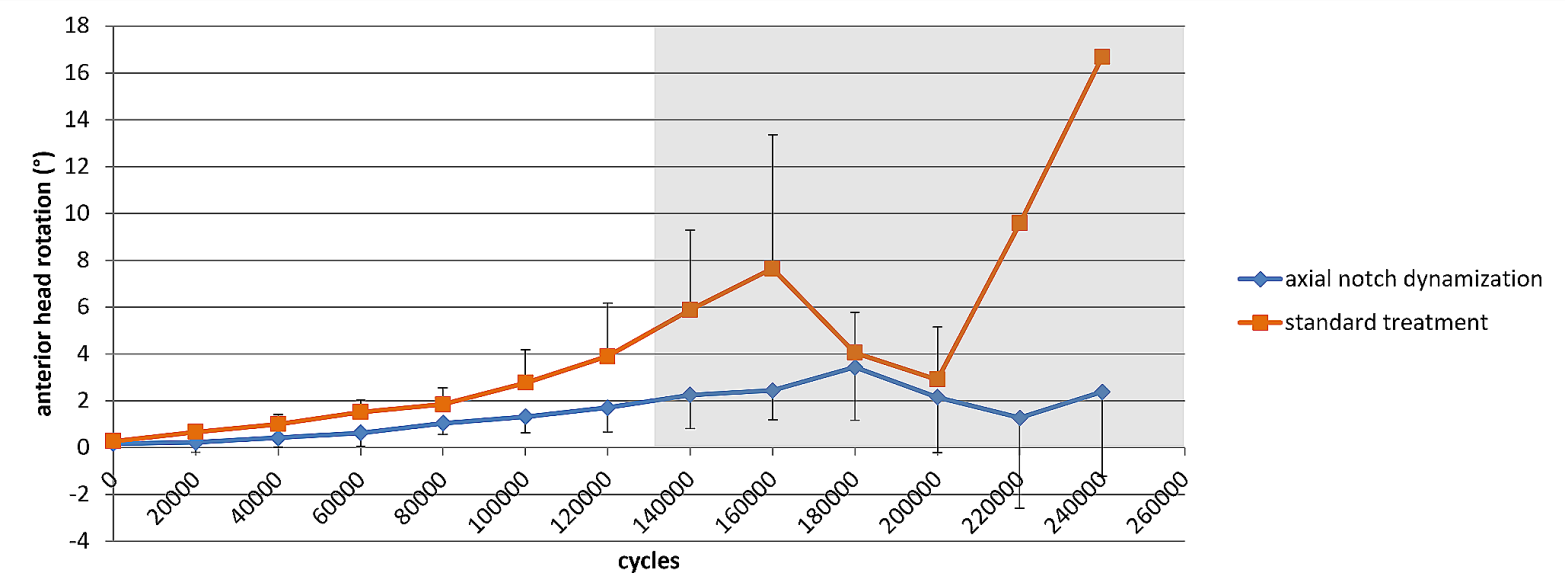
Anterior rotation of the femoral head fragment (mean with 95% confidence interval). All specimens survived up to 120,000 cycles. For load levels above 1100 N (grey area) only the remaining specimens that survived the respective load level are displayed

Rotation of the head fragment around the femoral neck axis occurred only towards anterior and resulted in consistently larger rotations for the standard treatment group (Fig. 5). At 1100 N anterior head rotation in the standard treatment group (3.9° [1.6°-6.2°]) was larger but statistically comparable to the axial notch dynamization group (1.7° [0.6°-2.8°]) (*p* = 0.14). For load levels above 1100 N, anterior rotation of the head fragment remained at a low level for the axial notch dynamized samples, while in the standard treatment group the rotation increased to a maximum of 16.6° in the remaining specimen.

### Failure modes

**Fig. 6:**
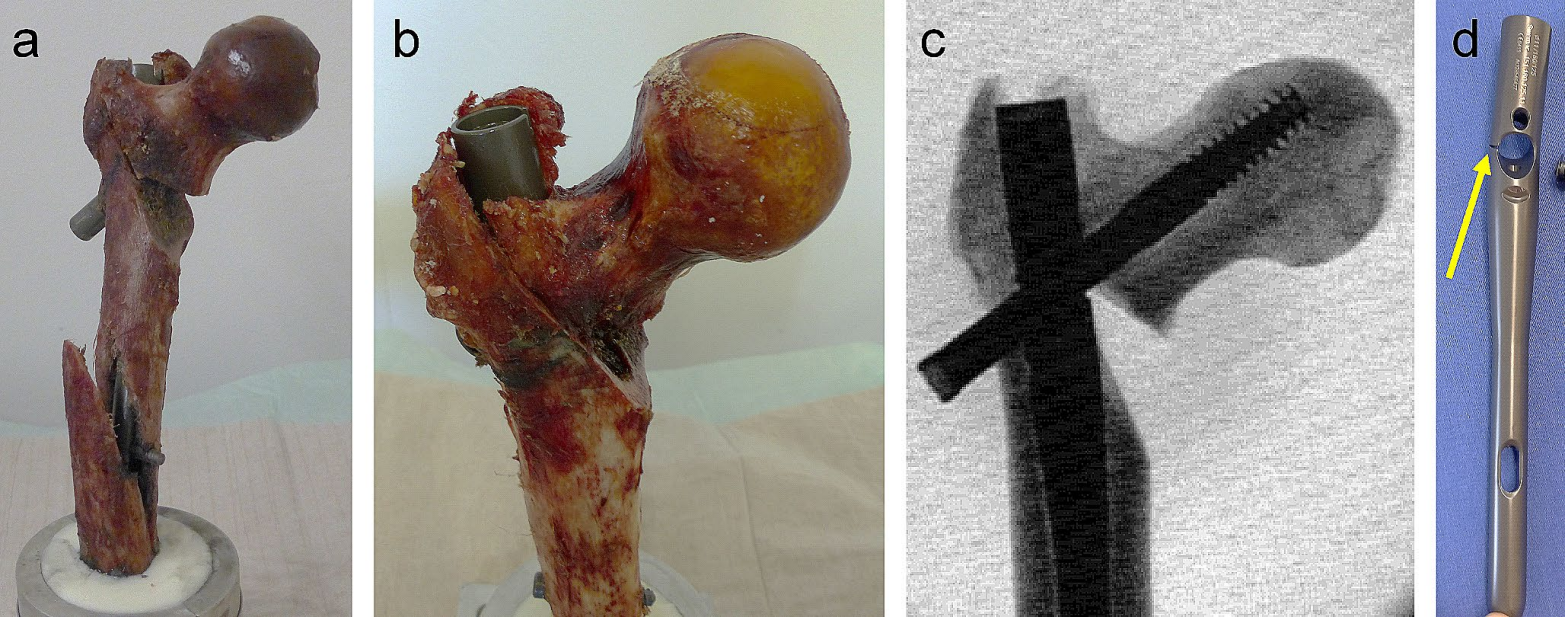
Examples of failure modes: shaft fracture **(a)**, subsidence of femoral head fragment **(b)**, varus collapse **(c)**, nail breakage indicated by the arrow **(d)**

Five out of 14 specimens failed due to fracture of the femur shaft (*n* = 1 axial notch dynamization, *n* = 4 standard treatment). In two specimens per group excessive axial subsidence (> 20 mm) of the head fragment terminated the mechanical test and in each group one varus collapse due to lag screw cut through occurred. In three specimens with axial notch dynamization, nail breakage occurred at its weakest point at the proximal aperture (Fig. 6).

## Discussion

In unstable pertrochanteric femur fractures treated by cephalomedullary nailing, fracture reduction and interfragmentary compression are of substantial importance to avoid non-unions or implant failures. Beside sliding of the construct along the axis of the lag screw, tapping the lateral cortex enables also a sliding along the femoral shaft axis. In this biomechanical study, we could confirm that axial notch dynamization facilitated compression of the fracture fragments. It also provided equivalent maintenance of mechanical stability during stepwise cyclic loading in terms of axial construct stiffness as well as interfragmentary motion compared to the standard treatment.

The concept of lateral cortical notching has already been reported by Tinner et al. in a clinical case report of an 87-year-old woman, having a subtrochanteric fracture treated by intramedullary nailing [6]. After non-union and revision surgery applying the lateral cortical notch technique, the fracture healed uneventfully [6]. This case report emphasized the importance of axial notch dynamization to promote enhanced interfragmentary compression and facilitate improved healing outcomes. Another study from Biber et al. reported that impaired bone healing in intertrochanteric fractures increases the risk of implant failure due to material fatigue [10]. The authors described a lateral notching procedure to enable effective distal dynamization in case of expected delayed union or non-union. A recent finite element study confirmed the beneficial effect of lateral cortical notching in unstable intertrochanteric fractures [12]. The authors found that tapping the lateral cortex below the lag screw reduces the stress at the bone-implant interface and thus, reduces the risk for implant failures. Beside the mentioned case report and finite element study, further clinical investigations with a primary focus on lateral notching in unstable pertrochanteric fracture fixation are required.

The dynamization concept of axial lateral notching is not only restricted to intramedullary implants. In 1991, an axial-compression screw-plate device for surgical treatment of pertrochanteric femur fractures was introduced by Medoff et al. [13]. This device allowed axial compression along the femoral shaft axis even after the fracture has settled. A modification of the Medoff sliding plate allowed sliding along the femoral shaft axis and optional sliding along the femoral neck axis, which offers biaxial dynamization [9]. This biaxial dynamization was found to provide a reliable treatment option and immediate weight-bearing was allowed [9]. In biomechanical investigations, biaxial dynamization offered comparable results in terms of femoral head displacements and appeared to provide improved load-sharing capabilities compared to a standard hip screw [14, 15]. Other extramedullary plating techniques provide dynamization solely along the axis of the lag screw; however, they offer a reliable treatment alternative and aim for correct anatomical alignment and a stable fracture fixation [16].

Standard treatment with a proximal femur nail had been shown to provide a stable construct as well [5]. In a similar study, the mean failure load for the Gamma3 nail (1430 N) and the Intertan nail (1640 N) showed comparable results to the present study [5]. Combining the advantages of standard proximal femoral nailing with applying the benefits of axial dynamization promises superior healing outcomes in more complex subtrochanteric or pertrochanteric fracture patterns. Further clinical studies are needed to prove this assumption.

In the present study, stiffness and interfragmentary movements were statistically analyzed up to a load of 1100 N, representing the highest load level of the weakest sample. This load level is in accordance to the median fatigue limit of the implant (1000 N), according to the manufacturer. Neither axial construct stiffness, nor interfragmentary movements showed any statistical differences between the two tested groups. Additional axial notch dynamization resulted in equivalent stability to the standard treatment approach.

A strength of this study was the physiological load protocol to simulate heel strike. During this phase of the gait cycle, axial load on the proximal femur is highest and the implant must provide highest stability [11]. The stepwise increasing axial load mimicked reduced weight-bearing immediately after surgery, as well as moderate and increased weight-bearing thereafter. A possible alternative to a stepwise increasing load protocol might be a protocol with continuously increasing load per cycle, potentially providing higher resolution for the detection of group differences. Construct failure after 191,708 ± 30,490 cycles (standard treatment) and 197,129 ± 45,087 cycles (axial notch dynamization) was estimated to be equivalent to 1–2 months postoperative movement [17]. This time interval covers the critical phase of either early construct failure or successful fracture healing [18].

In this study, in six out of seven specimens axial interfragmentary compression was enabled by dynamization along the shaft axis. Due to an even fracture surface resulting from the osteotomy, dynamization occurred already during the preconditioning cycles. However, given the typical irregularity of fracture surfaces in real pertrochanteric fractures, dynamization may occur during later courses of the healing process. Only in one specimen the axial notch dynamization was not triggered while loading. In this specimen with inhibited notch dynamization, and in further two specimens of the same group, the nail broke at its weakest portion at the proximal aperture [19]. Implant failure might be explained by excessive axial subsidence of the femoral head that caused considerable loading on the nail at the lag screw hole. In a previous study by Hoffmann et al., axial subsidence was found to be the major failure mechanism, primarily attributed to nail instabilities within the femoral shaft [5]. In the standard treatment group, no nail breakage occurred. However, the sample size of *n* = 7 is too low to draw a conclusive result from this failure mode.

Limitations of this biomechanical in-vitro study include the inherent weakness that in-vivo situations and healing processes cannot be realized. Furthermore, soft tissue was dissected and muscle forces were not considered. Although all implantations were carried out by a single surgeon and the fracture was created with the help of a sawing template to ensure reproducibility, it is likely that minor deviations in implant placement between specimens occurred due to inter-specimen variability [20]. Another limitation is related to the insertion of the lag screw. In 50% of the specimens the lag screw could not be inserted completely into the prepared screw hole. The correct screw length had been checked during drilling and the drill hole was free from debris. By using the option for an anti-rotation wire and by keeping the wedge fragment in place to further stabilize the head fragment during implantation, we could overcome these difficulties. Following this approach, a reproducible surgical technique for all specimens could be ensured.

In conclusion, the concept of axial notch dynamization showed equivalent maintenance of mechanical stability during stepwise cyclic loading compared to the standard treatment in this type of unstable pertrochanteric fracture. From a biomechanical perspective, lateral cortical notching can be recommended to enhance interfragmentary compression without compromising the overall mechanical stability of the fixation. Whether this additional dynamization along the femoral shaft axis fosters improved healing outcomes and reduces the risk of delayed union or non-unions, has to be investigated in clinical studies.

## Data Availability

No datasets were generated or analysed during the current study.

## References

[1] Mattisson L, Bojan A, Enocson A. Epidemiology, treatment and mortality of trochanteric and subtrochanteric hip fractures: data from the Swedish fracture register. BMC Musculoskelet Disord. 2018;19(1):369. 10.1186/s12891-018-2276-3. 30314495 10.1186/s12891-018-2276-3 PMC6186067

[2] Rapp K, Buchele G, Dreinhofer K, Bucking B, Becker C, Benzinger P. Epidemiology of hip fractures: systematic literature review of German data and an overview of the international literature. Z Gerontol Geriatr. 2019;52(1):10–6. 10.1007/s00391-018-1382-z. 29594444 10.1007/s00391-018-1382-z PMC6353815

[3] Garrison I, Domingue G, Honeycutt MW. Subtrochanteric femur fractures: current review of management. EFORT Open Rev. 2021;6(2):145–51. 10.1302/2058-5241.6.200048. 33828858 10.1302/2058-5241.6.200048 PMC8022017

[4] Panteli M, Mauffrey C, Giannoudis PV. Subtrochanteric fractures: issues and challenges. Injury. 2017;48(10):2023–6. 10.1016/j.injury.2017.09.001. 28992934 10.1016/j.injury.2017.09.001

[5] Hoffmann S, Paetzold R, Stephan D, Puschel K, Buehren V, Augat P. Biomechanical evaluation of interlocking lag screw design in intramedullary nailing of unstable pertrochanteric fractures. J Orthop Trauma. 2013;27(9):483–90. 10.1097/BOT.0b013e3182a1f54b. 23860133 10.1097/BOT.0b013e3182a1f54b

[6] Tinner C, Beckmann NA, Bastian JD. Lateral cortical notching in revision of a subtrochanteric fracture non-union with breakage of a Cephalomedullary nail. J Orthop Case Rep. 2020;10(6):5–8. 10.13107/jocr.2020.v10.i06.1852. 33489959 10.13107/jocr.2020.v10.i06.1852 PMC7815662

[7] Lim EJ, Shon HC, Cho JW, Oh JK, Kim J, Kim CH. Dynamic hip screw versus cannulated Cancellous Screw in Pauwels Type II or type III femoral Neck fracture: a systematic review and Meta-analysis. J Pers Med. 2021;11(10). 10.3390/jpm11101017. 10.3390/jpm11101017 PMC8541281 34683158

[8] Freigang V, Gschrei F, Bhayana H, Schmitz P, Weber J, Kerschbaum M, et al. Risk factor analysis for delayed union after subtrochanteric femur fracture: quality of reduction and valgization are the key to success. BMC Musculoskelet Disord. 2019;20(1):391. 10.1186/s12891-019-2775-x. 31470831 10.1186/s12891-019-2775-x PMC6717321

[9] Olsson O, Ceder L, Lunsjo K, Hauggaard A. Biaxial dynamization in unstable intertrochanteric fractures. Good experience with a simplified Medoff sliding plate in 94 patients. Acta Orthop Scand. 1997;68(4):327–31. 10.3109/17453679708996171. 9310034 10.3109/17453679708996171

[10] Biber R, Bail HJ, Stedtfeld HW. Lateral cortical notching in specific cases of delayed unions or nonunions after intertrochanteric and reversed fractures. Arch Orthop Trauma Surg. 2013;133(4):495–501. 10.1007/s00402-013-1683-z. 23329304 10.1007/s00402-013-1683-z

[11] Bergmann G, Bender A, Dymke J, Duda G, Damm P. Standardized loads acting in hip implants. PLoS ONE. 2016;11(5):e0155612. 10.1371/journal.pone.0155612. 27195789 10.1371/journal.pone.0155612 PMC4873223

[12] Hinz N, Stacenko K, Lutz C, Schulz AP, Wendlandt R. Lateral cortical notching facilitates dynamization of proximal femoral nailing - a finite element analysis. Injury. 2023;54(11):111009. 10.1016/j.injury.2023.111009. 37643944 10.1016/j.injury.2023.111009

[13] Medoff RJ, Maes K. A new device for the fixation of unstable pertrochanteric fractures of the hip. J Bone Joint Surg Am. 1991;73(8):1192–9. 1890120

[14] Olsson O, Kummer FJ, Ceder L, Koval KJ, Larsson S, Zuckerman JD. The Medoff sliding plate and a standard sliding hip screw for unstable intertrochanteric fractures: a mechanical comparison in cadaver femurs. Acta Orthop Scand. 1998;69(3):266–72. 10.3109/17453679809000927. 9703400 10.3109/17453679809000927

[15] Mahomed MN, Harrington IJ, Hearn TC. Biomechanical analysis of the Medoff sliding plate. J Trauma. 2000;48(1):93–100. 10.1097/00005373-200001000-00016. 10647572 10.1097/00005373-200001000-00016

[16] Bliven E, Sandriesser S, Augat P, von Ruden C, Hackl S. Biomechanical evaluation of locked plating fixation for unstable femoral neck fractures. Bone Joint Res. 2020;9(6):314–21. 10.1302/2046-3758.96.BJR-2019-0331.R1. 32637075 10.1302/2046-3758.96.BJR-2019-0331.R1 PMC7331880

[17] Schmalzried TP, Szuszczewicz ES, Northfield MR, Akizuki KH, Frankel RE, Belcher G, et al. Quantitative assessment of walking activity after total hip or knee replacement. J Bone Joint Surg Am. 1998;80(1):54–9. 9469309

[18] Norris R, Bhattacharjee D, Parker MJ. Occurrence of secondary fracture around intramedullary nails used for trochanteric hip fractures: a systematic review of 13,568 patients. Injury. 2012;43(6):706–11. 10.1016/j.injury.2011.10.027. 22142841 10.1016/j.injury.2011.10.027

[19] Li P, Zhang Z, Zhou F, Lv Y, Guo Y, Tian Y. Characteristics of intramedullary nail breakage in pertrochanteric femur fractures: a summary of 70 cases. J Orthop Surg Res. 2021;16(1):676. 10.1186/s13018-021-02826-3. 34789313 10.1186/s13018-021-02826-3 PMC8597261

[20] Gardner MJ, Silva MJ, Krieg JC. Biomechanical testing of fracture fixation constructs: variability, validity, and clinical applicability. J Am Acad Orthop Surg. 2012;20(2):86–93. 22302446 10.5435/JAAOS-20-02-086

